# SMART DIABETES HOSPITAL: CLINICAL IMPACT IN COMPLEX SURGICAL UNITS OF A TERTIARY HOSPITAL

**DOI:** 10.1007/s00592-024-02370-6

**Published:** 2024-09-06

**Authors:** Olga Simó-Servat, Judit Amigó, Ángel Ortiz-Zúñiga, Mónica Sánchez, Fátima Cuadra, Marcos Dos Santos, Alba Rojano, Maria José Abadías, Antonio Roman, Cristina Hernández, Rafael Simó

**Affiliations:** 1 Endocrinology and Nutrition Department, Vall d’Hebron University Hospital and Vall d’Hebron Research Institute, Barcelona, Spain; 2 Centro de Investigación Biomédica en Red de Diabetes y Enfermedades Metabólicas Asociadas (CIBERDEM), Instituto de Salud Carlos III (ICSIII), Madrid, Spain; 3 Health Services Research Group, Vall d’Hebron Research Institute and Vall d’Hebron University Hospital, Barcelona, Spain; 4 Universitat Autònoma de Barcelona, Barcelona, Spain

**Keywords:** Smart Diabetes Hospital, Specialized Diabetes Team, Management of hyperglycemia in the hospitalized patients, Length of stay of hospitalised patients with diabetes, Diabetes.

## Abstract

**Aim:**

To evaluate the impact of a proactive action of a specialized diabetes team (SDT) on different health outcomes in patients hospitalized in high complexity surgery units, including solid organ transplant surgical units, of a tertiary hospital.

**Methods:**

Nested case control study matched (1:1) by age and gender. The control group consisted of patients (*n* = 120) who were under the standard of care diabetes management admitted three months’ prior the cases. The cases were admitted in the same surgical units (*n* = 120) and were treated in the setting of the so called “Smart Diabetes Hospital” (SDH) consisting in a SDT that prioritized their actions through a digital map showing blood glucose levels obtained during the previous 24 h.

**Results:**

SDH implementation resulted in a significant reduction in both blood glucose levels (mean 162.1 ± SD 44.4 vs. mean 145.5 ± SD 48.0; *p* = 0.008) and hypoglycaemic episodes (19.7% vs. 8.4%: *p* = 0.002). Furthermore, a reduction of 3 days in the length of stay (LOS) was observed (15.6 ± 10.3 vs. 12.4 ± 6.0), which represents a significant cost-saving. Moreover, more new cases of diabetes were detected during the SDT period (2.5% vs. 6.7%, *p* = 0.04).

**Conclusion:**

SDH is effective in diabetes management and reduce LOS in complex surgical units.

**Supplementary Information:**

The online version contains supplementary material available at 10.1007/s00592-024-02370-6.

**STATEMENTS AND DECLARATIONS**:

The authors declare that they have no known competing financial interests or personal relationships that could have appeared to influence the work reported in this paper.

## AIMS

Hyperglycaemia is a very common condition in hospitalized patients due to the high prevalence of diabetes and the new onset or discovery of hyperglycaemia in the setting of hospitalization [1]. In this regard, it has been estimated that between 20 and 30% of patients in the hospital have diabetes [1].

Diabetes mellitus (DM) is associated with increased complications, increased length of stay (LOS) and higher mortality during hospitalization [2], thus leading to a significant increase in economic expenditure [3]. Therefore, the management of hyperglycaemia in hospitalized patients is not only meaningful for clinical purposes but also for socioeconomical reasons [4, 5].

Hyperglycemia confers an special vulnerability to hospitalized patients and there is evidence that it is associated with higher risk of complications during the admission and worse prognostic than patients without diabetes [1, 6]. In noncardiac surgery patients there is an association between blood glucose concentration and mortality; which is even more significant among patients without a history of diabetes compared with those with known diabetes before admission [7].

Although there are some guidelines on how to approach the treatment of in-hospital hyperglycaemia [8, 9], diabetes control remains a current challenge in hospitalized patients [2]. In fact, an audit carried out by the National Health Service in the UK detected that there were errors in insulin administration in up to 40% of patients treated with insulin during their admission [4]. For this reason, one early patient-centred strategy conducted by specialized diabetes team (SDT) aimed at adapting the established recommendations to the specific characteristics of the patient and the illness that led to admission is needed.

The SDT dedicated to diabetes inpatient care are composed mostly of a group of diabetes nurses supported by an experienced endocrinologist. They are a completely autonomous team, which provides early proactive assessment of patients with diabetes, which include daily treatment adjustment and diabetes education [10]. This is quite different from what happens in normal clinical practice where the endocrinology specialist or internist is consulted and they act in a reactive manner. However, little is known on the impact of SDT in the real world, and most of the studies are retrospective and focused on to medical units [3, 7, 13].

Perioperative hyperglycemia significantly increases the risk of infections and adverse cardiorenal events after general surgery, which leads to a higher length of stay in patients with diabetes compared to patients without diabetes admitted in the same surgical areas [7, 10]. The evidence of the effectiveness of SDT in improving glycaemic control and reducing the length of stay (LOS) in surgical units is limited to some pilot and retrospective studies [3, 7, 11, 12, 13], and to the best of our knowledge this has not been specifically examined in patients admitted in high complexity surgery and transplant units.

The aim of the study is to evaluate the impact of SDT action on different health outcomes in patients hospitalized in high complexity surgery units (including transplant surgical units) of a tertiary hospital. To prioritize the SDT action we have used a digital tool consisting in a glucose map showing the minimum and maximum blood glucose level during the previous 24 h. With this simple strategy that we have called “Smart Diabetes Hospital” (SDH), we have significantly reduced not only mean blood glucose levels and hypoglycaemic events, but also the LOS in these complex surgical units.

## METHODS

### 1.1 Model of care

In January 2022, an SDH was created to act promptly on the glycemic control of patients admitted in non-critical care units at Vall d’Hebron University Hospital, a tertiary referral medical centre in Spain. The selected patients for our action were people with DM who underwent a moderate or high complexity surgery or and solid organ (renal, hepatic or lung) transplantation including their complications. The surgical procedures apart from the solid organ transplantations were: limb revascularisation, surgery of acute biliary obstruction, pancreatic surgery, intestinal or stomach resection and total laryngectomy. The reason was the high cost associated with the treatment of these patients, which becomes particularly huge when there are associated complications during admission. Selected patients included those with pre-existing diabetes and those who develop hyperglycemia during the admission in the context of transplant medication, corticosteroids use or pancreatectomy. Hyperglycemia in hospital status was defined as ≥ 2 random capillary blood glucose (BG) measurements ≥ 200 mg/dL.

Before the unit was settled, a specific digital tool was designed to identify and prioritized those patients requiring intervention by SDT. Once the hospitalisation nurses have introduced the blood glucose (BG) readings into the computer program as they usually do, data is transferred to the “blood glucose map”. All patients with BG determination are included in this map, showing their minimum and maximum BG during the previous 24 h. A colour legend was also designed so that BG readings above or below the desired glucose range were easily identified. This map is updated three times a day with the new BG of the hospitalised patients including new admissions.

Our SDT was formed by a diabetes nurse educator and an endocrinologist of the Technology Diabetes Unit. The SDT was in charge of the diabetes evaluation and its management from admission to discharge. Clinical evaluation of the patient and adjustment of insulin treatment was performed daily based on current recommendations [9]. An advanced programme of nursing education was also done according to the patient’s needs. Finally, before discharge the best route for diabetes follow-up was selected according to the characteristics of the patient and the preferences of the family and/or caregivers. For those patients with a recent diabetes onset and all cases in whom a close follow up was needed, an appointment in the Diabetes Day Hospital (DDH) was scheduled within 1–2 weeks after discharge. The SDT operates from Monday to Friday from 8 h to 17 h am.

The study was performed in accordance with the Declaration of Helsinki.

### 1.2 Study design

In order to have similar groups to compare the effect of our intervention a nested case control study was planned. The inclusion criteria were patients admitted to moderate and high complexity surgery units, including solid organ transplant surgical units, who had had a hospital stay of more than 4 days and with a firmly established diagnosis of diabetes. We consider that less than 4 days is a short admission period to evaluate the real impact of SDH on the study outcomes and, therefore, these patients were excluded. A total of 120 patients who fulfilled the inclusion criteria admitted from January to March 2022 were evaluated by SDT (intervention group). The control group were patients hospitalised in the same floors from October to December 2021that met the inclusion criteria, and matched 1:1 based on age (± 2 years) and sex with the intervention group. The control group was recruited before the SDH started, but there were no changes in protocols for treating hyperglycemia or other external factors that could influence the diabetes management. Treatment of diabetes during that period was performed by the medical teams responsible for the hospitalisation and in some cases by the endocrinologist when a consultation was performed.

### 1.3 Data collection and statistical analysis

In order to evaluate the impact of the SDH we compared the outcomes listed below comprising 3 months prior and 3 months after starting the intervention program with SDT. We collected different outcomes related to the following items: **(1) Hospital admission**: acute pathology that motivates admission, medical or surgical complications (defined as any adverse outcome that takes place after surgery is performed), length of stay; **(2) Pre-existing diabetes characteristics**: metabolic control (HbA1c), treatment (insulin therapy or oral agents); associated diabetic complications, previous follow-up **(3) Diabetes management during admission**: treatment, BG readings and hypoglycaemic episodes (defined as BG < 70 mg/dl); **(4) Patient destination after hospital discharge.**

The glucometric measure obtained from the BG readings is the mean of all glucose values taken for a patient during admission.

STATA programme was used for the statistical analysis. Categorical variables are presented as percentages. For the quantitative variables, the mean and standard deviation is displayed. Statistical significance was accepted at *p* < 0.05. To assess differences between groups, the Chi-square test for qualitative variables, and the T-Student for the quantitative variables was used.

## RESULTS

The baseline characteristics of patients and variables related to diabetes in both groups (patients with and without the support of SDH) are shown in Table 1. As per protocol, we did not find any significant difference in age and gender. Both groups presented similar HbA1c levels and percentage of patients under treatment with insulin. In addition, we did not observe significant differences between groups in the frequency of diabetes related complications with the exception of peripheral arteriopathy, which was more frequent in those patients receiving the support of the SDH.

**Table 1:** Baseline characteristics of patients and main variables related to diabetes

	Standard of Care group (*n* = 120)	SDH group (*n* = 120)	*p*
**Age (years**,** mean** ± **SD )**	71.0 ± 11.9	70.2 ± 11.9	0.59
**Sex (%)**	70 Men 30 Women	70 Men 30 Women	0.59
**Type of DM (%)**	94.1 DM2 2.5 DM1 3.4 Others*	90 DM2 3.6 DM1 6.4 Others*	0.11
**HbA1c (%)**	7.2 ± 1.7	7.1 ± 1.6	0.93
**Treatment with insulin (%)**	46.6	50.9	0.75
**Diabetic complications (%)**: **- Retinopathy (%)** **- Nephropathy (%)** **- Polineuropathy (%)** **- Coronary heart disease (%)** **- Cerebrovascular disease (%)** **- Peripherical arteriopathy (%)**	71.2 14.2 30.8 10.2 22.5 13.3 21.6	65 21.6 31.7 21.7 12.4 10 37.3	0.31 0.13 0.89 0.07 0.50 0.44 0.01

*Others: Secondary to pancreatectomy and PTDM

During admission, all patients were treated with subcutaneous insulin, most of them with basal plus bolus in sliding scale regimen. The comparison between groups in terms of glucometric variables and general outcomes is shown in Table 2. We found an optimization of glycemic control in patients with the support of SDT in the setting of SDH in comparison with standard of care. In this regard we observed a significant reduction in the mean of capillary blood glucose levels during admission 162.1 ± 44.4 vs. 145.5 ± 48 (p = 0.008) without increasing the rate of hypoglycemia. In fact, a significant reduction of hypoglycemic episodes was found (19.7% vs. 8.4%: p = 0.002). Furthermore, a reduction from 15.6 ± 10.3 vs. 12.4 ± 6.0 in the LOS was obtained, which represent a cost saving of 218.880 € in the period of the study (3 months). This was calculated based just on general cost (not including specific treatments) for a day of admission in this type of surgical unit in a tertiary hospital of Catalonia Health System (acronym ICS meaning in Catalan *“Institut Català de la Salut*”). Moreover, with the SDT intervention hyperglycemia was detected more often (2.5% vs. 6.7%, *p* = 0.04).

**Table 2:** Comparison between groups (Standard of care vs. SDH) in terms of glycemic parameters and general outcomes

	Standard of Care group *N* = 120	SDH group *N* = 120	*p*
**Median of the blood glucose readings during admission (mg/dl**,** mean ± SD)**	162,1 ± 44,4	145,5 ± 48,0	0,008
**Hypoglycemic episodes (%)**	19,7	8,4	0,03
**Complications during admission (%)**	48,3	49,2	0,90
**Mortality (%)**	4,2	3,4	0,73
**LOS (days**,** mean ± SD)**	15,6 ± 10,3	12,4 ± 6,0	0,004

We have identified two main groups of patients in whom the SDT intervention had the most significant impact. One is he subgroup of patients under treatment with insulin previous to the admission. In this subgroup the hypoglycemic rate was higher and SDT intervention significantly reduced this adverse effect (25.9% vs. 9.01%, *p* = 0.021). The second subset of patients were those admitted for transplantation or related comlications (mainly acute or chronic rejection). In these patients a decrease in readmissions was observed (68,4% vs. 7,1%, *p* < 0.01), and this was probably attributed to a greater attendance at DDH.

We did not find any significant differences in complications related to surgery, due to cardiorenal impairment, infections, thrombosis/hemorrhage or related to graft being in both groups the more prevalent one the infectious one. Furthermore, patients allocated to SDH presented less mortality rate but without reaching statistically significance (3.4% vs. 4.2%; *p* = 0.73).

Finally, the patient destination after discharge was different between the two groups. More proportion of patients allocated to SDH were followed-up by the SDT at DDH and by the endocrinologists of our engagement area in comparison with the control group (26.8% vs. 13.6%; *p* = 0.01).

## CONCLUSIONS

The diabetes control in the hospital, and in particular in surgical units, is a challenge for all the health care systems. In the setting of a tertiary hospital, the current manner to proceed by the Endocrinologists or internal Medicine specialists is to await a consultation from the surgical team aimed at addressing the diabetes control, which is usually received too late and result in an increase of LOS. This is because, apart from the necessary treatment changes and further adjustments, most of patients have not the sufficient knowledge on diabetes management to guarantee a self-care at home after discharge. It should be noted that the vast majority of patients are discharged from these surgical units under treatment with insulin in basal bolus regimen but a high percentage (around 50% in our series) were under non-insulin treatment before admission. This represents a new and a stressful situation for the patients, their families and/or caregivers and in particular if we consider that the mean age is generally advanced (over 70 years in our series).

On this basis we have evaluated the initial impact of an SDH in moderate and high complexity surgery units (including transplant surgical units) of a third level University Hospital. To the best of our knowledge, no previous studies on this topic including this type of patients have previously been reported. The SDH consist in the combination of a SDT which is supported by a digital tool consisting in a glucose map showing the minimum and maximum blood glucose level during the previous 24 h. The periodic monitoring of the blood glucose levels in the surgical units permit us to be proactive and prioritize the patients who are requiring immediate attention for optimizing their metabolic control, without any requirement of consultation from the surgical team. In addition, a specific diabetes education programme is implemented. With this strategy we have had a reduction of LOS of 3,4 days in these complex surgical units and a significant improvement in glycemic control during admission, as well as a significant reduction of hypoglycemic episodes. Obviously, the LOS reduction represents a very important saving for the hospital cost, which make the model sustainable and eventually extensible to other surgical and medical units. Moreover, it should be stressed that patients included in the surgical units selected for this study are those patients in which the healthcare system has already made a large investment of resources.

In addition, SDH allow us to detect more new cases of diabetes during admission, which is meaningful for early treatment and significantly contribute to avoid a delay in the discharge. This is particularly important in the post-transplant diabetes (PTDM) defined as the occurrence of diabetes in a previously non-diabetic person after solid organ transplantation. This is a serious and common complication following solid organ transplantation and increases the risk of infection and mortality rates [14–16]. There is strong evidence suggesting that kidney transplant recipients who developed PTDM are at a two- to three-fold increased risk of fatal and nonfatal cardiovascular disease events as compared with nondiabetic patients (17–18). The development of PTDM has also been shown to be associated with an adverse impact on patient survival and an increased risk of graft rejection and graft loss, as well as an increased incidence of infectious complications [16–19]. The early identification and treatment just after transplantation might have beneficial consequences in terms of reduce complications and graft rejection but larger studies are needed to confirm this hypothesis.

Another important point is the impact of the SDH on the patient journey after discharge. In this regard, it should be noted that before discharge the best route for diabetes follow-up was selected according to the characteristics of the patient and the preferences of the family and/or caregivers by the SDT. In addition, a programme of diabetes education was done according to the patient’s needs and an appointment to the DDH was facilitated if required. As a result of these action around 12% of patients were reallocated to routine control by an endocrinologist instead of general practitioner.

The strength of this study is that the comparison between standard of care and the SDH was performed in the same hospital units and with the same personnel, thus avoiding any bias related to type of patients and hospital conditions. The main limitation is the relatively small recruitment period (3 moths) which result in not very large sample size sample. This limitation could play a role in the lack of significant reduction in surgical complications and mortality. Another limiting point is that SDT only operates during working days. This probably impacted the continuity of care and also delayed the SDT intervention in the case of patients admitted during the weekend. Therefore, in the event that SDH was also working the weekend the results would probably have been even better. Finally, although the degree of satisfaction of patients included in the SDH program was excellent we cannot compare with the results obtained in the standard of care because in this later the appropriate questionnaires were not implemented.

Our results provide evidence that the monitoring of glycemic control is cost-benefit in the real world in those patients requiring high complexity surgery or a solid organ transplantation. This finding paves the way to the use of continuous glucose monitoring (CGM) at least in this subset of population. The use of CGM in these selected patients would give us a more comprehensive picture of the metabolic control by providing important metrics on glycemic variables. Therefore, it is expected that use of CGM will improve the results herein reported.

In conclusion, a pro-active attitude aimed at improving glycemic control and detecting new cases of diabetes by combining SDT and digital glycemic maps is effective in shortening the hospital stay of patients admitted in complex surgical units (including solid organ transplantation). This is important because, apart from saving costs, a reduction of surgery complications or graft rejection in this particularly vulnerable population could be achieved. However, further studies with large sample size to confirm this hypothesis are needed.

## Electronic supplementary material

Below is the link to the electronic supplementary material.

Supplementary Material 1
